# CiFi: Accurate long-read chromatin conformation capture with low-input requirements

**DOI:** 10.1101/2025.01.31.635566

**Published:** 2025-02-05

**Authors:** Sean P. McGinty, Gulhan Kaya, Sheina B. Sim, Renée L. Corpuz, Michael A. Quail, Mara K. N. Lawniczak, Scott M. Geib, Jonas Korlach, Megan Y. Dennis

**Affiliations:** 1 Genome Center, MIND Institute, and Department of Biochemistry & Molecular Medicine, University of California, Davis, CA 95616, USA.; 2 U.S. Department of Agriculture, Agricultural Research Service, U.S. Pacific Basin Agricultural Research Center, Tropical Pest Genetics and Molecular Biology Research Unit, Hilo, HI 96720, USA.; 3 Wellcome Sanger Institute, Hinxton CB10 1SA, UK.; 4 Pacific Biosciences, Menlo Park, CA 94025, USA.

**Keywords:** Chromatin conformation capture, long-read sequencing, HiFi, CiFi, PacBio, genome assembly, scaffolding, genome organization

## Abstract

By coupling chromatin conformation capture (3C) with PacBio HiFi long-read sequencing, we have developed a new method (CiFi) that enables analysis of genome interactions across repetitive genomic regions with low-input requirements. CiFi produces multiple interacting concatemer segments per read, facilitating genome assembly and scaffolding. Together, the approach enables genomic analysis of previously recalcitrant low-complexity loci, and of small organisms such as single insect individuals.

## MAIN

Hi-C, a sequencing technique that assays 3C genome wide^1^, provides insights into chromatin organization and *cis*-regulatory interactions. The method relies on *in situ* proximity ligations produced by endonuclease cutting of cross-linked chromatin in cells that results in preferential binding of genomic fragments positioned physically near to each other in the nucleus. Given that a majority of reads represent intrachromosomal interactions, Hi-C is also an effective tool for haplotype phasing and genome assembly^2,3^.

Short-read sequencing is classically employed in Hi-C with paired-end reads representing two interacting regions. While effective across a majority of the genome, short reads can fail to map uniquely across repetitive and low-complexity regions. Further, higher-order chromatin interactions that exist within the nucleus are difficult to parse when looking through a pairwise lens. Multi-contact 3C (MC-3C)^4^ and Pore-C^5^ have recently been developed that combine 3C with long-read sequencing (PacBio and nanopore, respectively) enabling a more complete picture of proximally-interacting concatemers. While these approaches are effective in disentangling three-dimensional (3D) genome folding at the allele level^6,7^, both require significant starting material (e.g., 10 million cells for Pore-C) that have made them inaccessible to certain applications and organisms.

**Figure 1.**
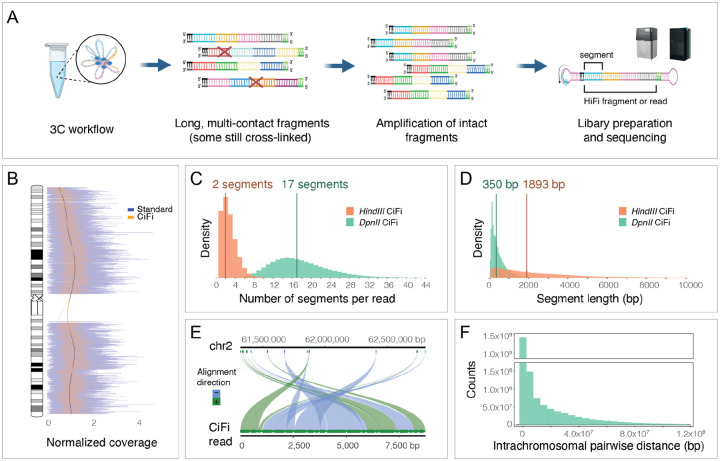
CiFi produces unbiased 3C concatemer long reads improving genomic mapping of segments. **(A)** Overview of the CiFi approach. **(B)** Normalized read coverage comparison of Sequel II data for *DpnII* 3C libraries generated without (Standard) and with the amplification-protocol (CiFi) for GM12878 across human chromosome 1. **(C)** Segment number distribution per HiFi read and **(D)** length distribution of concatemer segments for *DpnII* and *HindIII* CiFi libraries, with medians indicated. **(E)** An example of mapping positions of individual segments across human chromosome 2 from a single *DpnII* CiFi read. **(F)** Histogram of pairwise intrachromosomal distances between CiFi segments from the same HiFi read.

To overcome this challenge, we sought to optimize 3C coupled with PacBio HiFi sequencing. Testing library preparations on the human GM12878 lymphoblastoid cell line (LCL) with *DpnII* restriction endonuclease (as described for Pore-C^5^), we observed low sequence yields (10–20% of standard Sequel II runs; Table S1). While SMRT Cell loading was as expected, polymerase read lengths were short (mean 33.4 kbp) and showed a low abundance of HiFi reads (9.8% of polymerase reads; 3.46 Gbp at mean read length 7.24 kbp; Table S1). Repeating the experiment on LCLs using the MC-3C protocol yielded a similarly low HiFi yield, which is in line with published work^4,7^ (Tables S1 and S2). Hypothesizing that residual cross-links remained on the DNA that prevent productive sequencing, following 3C we implemented a genome-wide amplification-based protocol designed for challenging samples^8^, using a high-fidelity PCR enzyme to enrich for uncross-linked molecules ahead of sequencing (Figure 1A). This dramatically increased raw sequence yields and read lengths to standard sequencing performance (mean 105.3 kbp) and conversion to HiFi data (49.3% of polymerase reads; 30.6 Gbp at mean read length 9.35 kbp and median read quality value (QV) 38; Table S1). The previous study^8^ observed limited PCR biases; this was also the case for our GM12878 *DpnII* 3C library with 1.8% of our data representing PCR duplicates and no obvious dropouts evident when comparing read coverage with and without amplification (Figures 1B and S1).

To investigate the effects of multi-contact segment lengths and their resulting resolution of repetitive genomic regions, we performed 3C with *DpnII* (4 cutter) and *HindIII* (6 cutter), respectively, followed by amplification, size selection (>5 kbp), and Revio sequencing (Figure S2). From the resulting HiFi reads (median lengths of 8.0 kbp; Table S1), subsequent *in silico* digestion produced a median of 17 segments at 350 bp for *DpnII* and 2 segments at 1,893 bp for *HindIII* (Figures 1C–D and Table S3). We next mapped against the human reference genome (T2T-CHM13_v2^9^) and converted segments within each HiFi read into paired interactions (Figure 1E). For example, a single *DpnII* CiFi read with 17 segments represents 136 pairwise interactions, equivalent to 272 Illumina read pairs. Applying this to each CiFi datasets resulted in 2.1 billion interactions for *DpnII* and 38.5 million interactions for *HindIII*. Pairwise interactions showed the expected 3C decay with increasing distance (Figure 1F) and spanned all length scales, going as far as >100 Mbp (the average size of a human chromosome).

**Figure 2.**
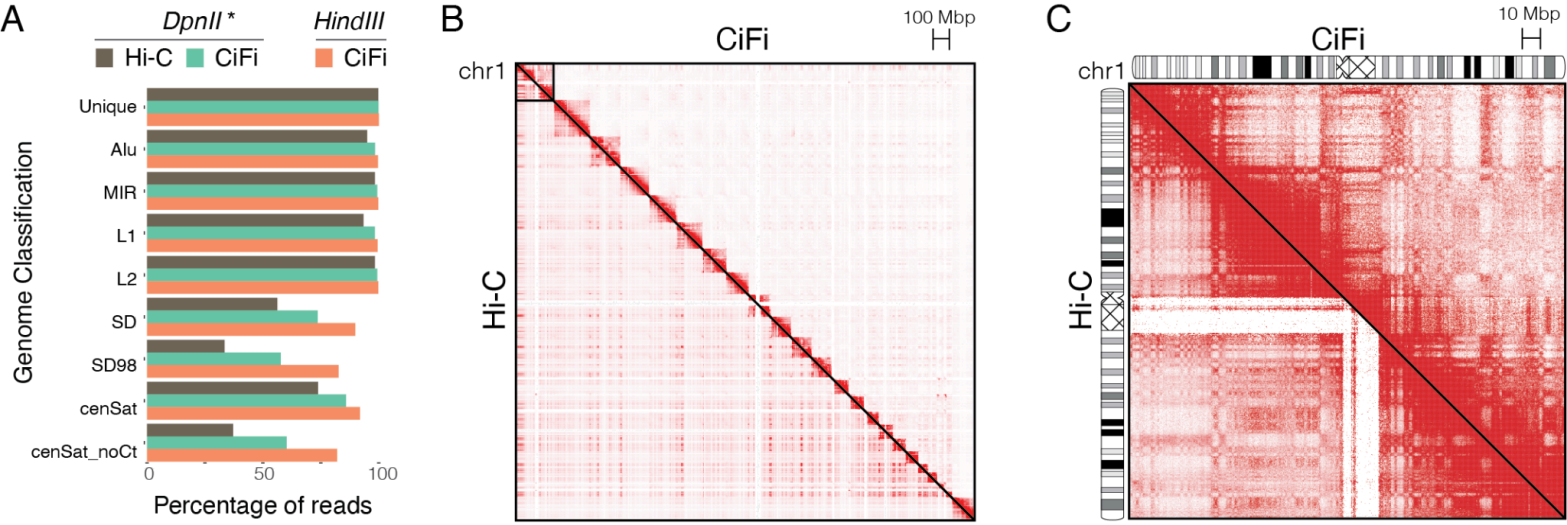
Comparisons of chromatin contacts for human LCL GM12878 between CiFi and Hi-C. (**A**) Percent of reads with MAPQ cutoff of one or higher for Hi-C with Illumina^10^ and CiFi across different repetitive genome classifications. *DpnII libraries for CiFi (x-axis) and Hi-C (y-axis) were subsequently used to generate pairwise interaction maps (2.5 Mbp resolution) **(B)** genomewide (not normalized); and **(C)** across chromosome 1 (normalized using the Knight-Ruiz algorithm^11^). Cytogenetic bands and the centromere (criss-cross pattern) are depicted on each chromosome 1. Increasing red shading corresponds to higher numbers of paired reads.

Comparing mappings of CiFi segments with 101-bp paired-end Illumina Hi-C sequencing reads—generated as a standard resource for GM12878^10^—showed better representation across non-“unique” genome space, including short interspersed nuclear elements (SINEs) *Alu* and *Mir*, long interspersed nuclear elements (LINEs) *L1* and *L2*, segmental duplications at both 90% (SD) and 98% (SD98) identity, and centromeres with and without the centromeric transition (CT) regions (Figures 2A and S3). Improvements were most evident across SDs and centromeres, with only 33–37% of Illumina Hi-C reads exhibiting a MAPQ cutoff ≥1 compared with 83–89% of CiFi with *HindIII* reads. Using pairwise data to generate genome-wide contact matrices (Figures 2B and S4), we found an increased proportion of intrachromosomal interactions for *DpnII* CiFi (77.2%) versus Illumina Hi-C (65.7%). The two datasets largely correlate across chromosomes at 2.5 Mbp resolution (r^2^ = 0.886), with unique regions more highly correlated (r^2^ = 0.925) versus SDs and centromeres (r^2^ = 0.687) due to the reduced read coverage of Hi-C (Figure 2C and S5). Topologically-associating domains also exhibited concordance (MoC = 0.818) between methods across chromosome 1 and chromosome 2 (Figure S6), with notable differences at SDs. Together, CiFi and Hi-C similarly represent genome organization across a majority of the human genome, with CiFi providing notable improvements across repetitive and complex regions.

**Figure 3.**
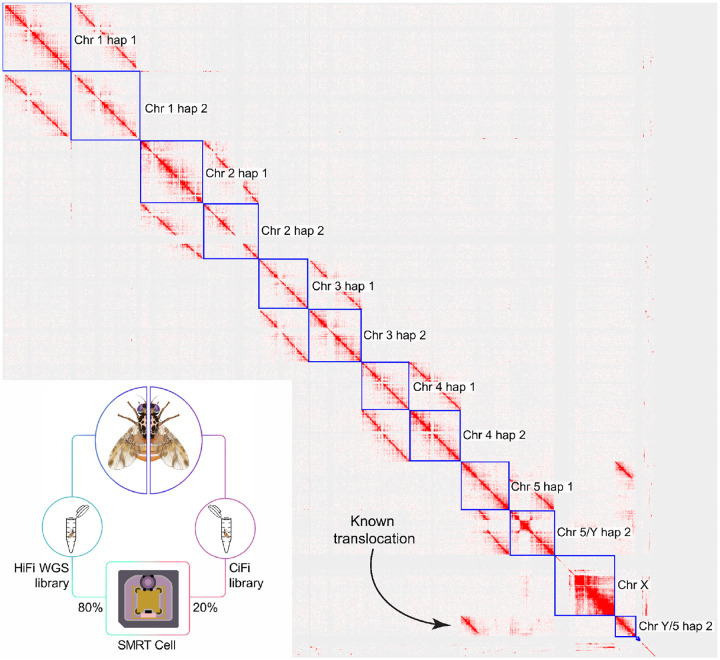
*De novo* assembly of a single Mediterranean fruit fly (*C. capitata*) using a combination of HiFi and CiFi sequencing. Inset: workflow for generating a HiFi WGS and CiFi library from the same individual. CiFi contacts were used to scaffold the HiFi-based contig assembly into a chromosome-scale, phased diploid assembly.

To explore the benefits of lower input requirements, we first scaled down GM12878 *DpnII* CiFi samples by over 100-fold, from 10 million cells (~60 μg of DNA input) to 62,000 cells (~370 ng), resulting in consistent sequencing metrics and contact matrices across all starting amounts (Tables S1 and S4). Moving beyond human cell lines, we next applied *DpnII* CiFi to a single *Anopheles coluzzii* mosquito (~250 ng starting input^12^) and generated 2.37 million HiFi reads and 21.1 million segments of median length 509 bp (Tables S1 and S3). From this, we successfully assayed pairwise chromatin interactions across the 263 Mbp reference genome (AcolN3) (Figure S7). Third, we leveraged CiFi to assemble the ~600 Mbp genome of a single Mediterranean fruit fly, *Ceratitis capitata*, by generating PacBio HiFi whole-genome sequencing (WGS) and CiFi reads from opposite halves of the same male individual split laterally (Figure 3, Table S5). We successfully tested both the combining of HiFi and CiFi libraries in a single Revio sequencing run, and the barcoding and pooling of several CiFi libraries from different samples ahead of sequencing (Table S1). Using *HindIII* CiFi reads to both phase within^13^ and scaffold across contigs^14^ resulted in a chromosome-scale, diploid assembly (2n = 12) (Figure 3). Adjusted QVs of the phased, chromosome-scale scaffolds ranged from 41.4–58.1^15^ with 96.7–99.3% complete Diptera BUSCO values^16^ (Table S5). Mapping CiFi fragments as paired-end reads back to the assembly showed the expected intrachromosomal contacts, and interactions between chromosomes 5 and Y, marking a known translocation characteristic of the strain used here^17^. Subsampling CiFi reads used to scaffold this assembly revealed that a minimum of 70,000 CiFi reads, resulting in approximately 300,000 proximity ligated pairs (equivalent to 1.5× coverage), were necessary for chromosome-scale scaffolding that is on par with the curated reference^18^ (Figure S8).

Highlighting limitations of the current CiFi approach, the use of restriction endonucleases has the potential to introduce biases in the regions assayed with certain loci naturally depleted for cutting sites; this can be mitigated by choosing alternative restriction sites or employing non-specific endonucleases^19,20^, representing lines of future improvements. In addition, while we show that CiFi enables haplotype separation, phasing, and assembly scaffolding, improved computational approaches^21^ that take full advantage of the higher-order multi-contact interactions inherent in CiFi reads can likely further enhance the resolving power of the approach.

In summary, CiFi enables efficient PacBio HiFi sequencing of 3C libraries from diverse species and sample types, and allows for high-quality, haplotype-resolved, chromosome-scale *de novo* genome assemblies with data from one sequencing technology, and, if desired, from a single sequencing run. A typical ~8 kbp CiFi read comprises multi-contact segments ranging in size from ~350 bp to 2 kbp that provides genome-wide chromatin contexts, including highly repetitive and low-complexity regions. We anticipate that the low-input requirement will expand the application of 3C with long-read sequencing to many single small organisms, as well as other samples, including isolated cell types and disease specimens such as tumor biopsies.

## METHODS

We developed an optimized protocol for multi-contact, long-read Hi-C library preparation with low DNA input, enabling efficient and accurate sequencing on the PacBio platform. Our approach overcomes traditional high-input limitations and enhances the resolution of 3D chromatin interactions. For detailed, step-by-step procedures, including reagent volumes and amendment to protocols, please refer to the Supplementary Information and Table S6, respectively.

### Cell culture and cross-linking

The GM12878 cell line was cultured in RPMI 1640 medium supplemented with 15% fetal bovine serum and 1% penicillin-streptomycin. Cells were maintained at 37°C in a CO_2_ incubator and tested biannually for mycoplasma contamination using a PCR detection kit (abm, Cat# G238). For cross-linking, 5–10 million cells were washed three times with cold phosphate-buffered saline (PBS) and resuspended in PBS containing 1% formaldehyde (EMD Millipore, Cat# 818708). Following a 10-minute incubation at room temperature, glycine was added to a final concentration of 125 mM to quench the reaction. Cells were incubated for an additional 5 minutes at room temperature and 10 minutes on ice, then centrifuged at 500×g for 5 minutes at 4°C. The pellet was washed with PBS, snap-frozen in liquid nitrogen, and stored at −80°C.

### Restriction enzyme digestion

The cell pellet was resuspended in 50 μL of protease inhibitor cocktail (Sigma Aldrich, Cat# P8340) and 500 μL of cold permeabilization buffer (10 mM Tris-HCl pH 8.0; 10 mM NaCl; 0.2% IGEPAL CA-630). After a 15-minute incubation on ice, cells were centrifuged and resuspended in 300 μL of chilled 1.5× digestion reaction buffer compatible with the chosen restriction enzyme (NEB). Chromatin was denatured by adding SDS to a final concentration of 0.1% and incubating at 65°C for 10 minutes with gentle agitation. SDS was quenched with Triton X-100 to a final concentration of 1%. Permeabilized cells were digested with 1 U/μL of *DpnII* or *HindIII* (NEB) in a final volume of 450 μL, maintaining a 1× digestion buffer concentration. The mixture was incubated at 37°C for 18 hours with gentle mixing to prevent condensation inside the tube.

### Proximity ligation and reverse cross-linking

Following digestion, the restriction enzymes were heat-inactivated at 65°C for 20 minutes for *DpnII* and at 80°C for 20 minutes for *HindIII*, then immediately placed on ice. T4 DNA ligase (NEB M0202L) and ligation buffer were added to the mixture, which was then incubated at 16°C for 6 hours with gentle rotation. To degrade proteins and reverse cross-links, Proteinase K (Thermo Fisher Scientific, Cat# 25530049), SDS, and Tween-20 were added. The sample was incubated at 56°C for 18 hours with intermittent mixing. DNA was purified using phenol-chloroform extraction and ethanol precipitation, and then resuspended in TE buffer. To evaluate experimental efficiency, undigested, digested, and ligated DNA products were analyzed via agarose gel electrophoresis (Figure S9).

### Size selection and quality control

For libraries prepared using *DpnII*, size selection was performed using AMPure PB beads (PacBio) at a 0.45× ratio according to the manufacturer’s protocol. DNA size distribution was assessed using Femto Pulse automated pulsed-field capillary electrophoresis, confirming an expected size range suitable for SMRTbell library preparation (Figure S10A). For *HindIII*, additional DNA shearing was required before size selection due to the longer fragment sizes. The DNA was sheared to approximately 10 kbp using a g-TUBE (Covaris, 520104), following the manufacturer’s protocol, and then followed by size selection with AMPure PB beads (PacBio) at a 0.45× ratio. The DNA size distribution was similarly confirmed using Femto Pulse analysis.

### SMRTbell library preparation from low DNA input

Single-strand overhangs were removed by treating the DNA with DNA Prep Buffer, NAD, DNA Prep Additive, and DNA Prep Enzyme, followed by incubation at 37°C for 15 minutes. DNA damage was repaired using DNA Damage Repair Mix v2 (PacBio) with a 30-minute incubation at 37°C. End repair and A-tailing were performed by adding End Prep Mix and incubating at 20°C for 30 minutes and then at 65°C for 30 minutes. Adapters were ligated to the repaired DNA using diluted Amplification Adapters (PacBio), Ligation Mix, Ligation Additive, and Ligation Enhancer, with incubation at 20°C for 60 minutes. The SMRTbell library was purified using SMRTbell beads (PacBio) and eluted in EB buffer. Library concentration was measured using the Qubit dsDNA HS assay (Thermo Fisher Scientific).

### Library amplification via PCR

To amplify the library, PCR reactions were set up using KOD Xtreme hot-start polymerase (Sigma, Cat. No. 71975-M) due to its high fidelity and efficiency with low DNA input samples. Each reaction contained 2× Xtreme buffer, 2 mM dNTPs, sample amplification PCR primer, purified SMRTbell library, and polymerase. The PCR conditions were as follows: initial denaturation at 94°C for 2 minutes; 13 cycles of denaturation at 98°C for 10 seconds, annealing at 60°C for 30 seconds, and extension at 68°C for 10 minutes; and a final extension at 68°C for 5 minutes. PCR cycle numbers were optimized based on initial template concentration to achieve the desired yield. Amplified DNA was purified using SMRTbell beads and quantified using the Qubit dsDNA HS kit. DNA size distribution was confirmed with Femto Pulse electrophoresis, ensuring a size mode of 8–10 kbp (Figure S10B).

### Final library preparation and sequencing

The amplified DNA underwent a second round of DNA damage repair, end repair, A-tailing, and adapter ligation using Overhang Adapter v3 or barcoded adapters for multiplexing (Figure S10C). The final SMRTbell library was purified using SMRTbell beads and assessed for concentration and size distribution. Size selection was performed using the BluePippin system (Sage Science) to enrich for fragments larger than 5 kbp for GM12878 *DpnII* (Figure S10D) and larger than 8 kbp for the Mediterranean fly. For GM12878 with *HindIII* and mosquito, an alternative size selection method with diluted AMPure PB beads was performed. The initial optimization of the CiFi protocol for GM12878 with *DpnII* used the SMRTbell 2.0 kit. CiFi libraries for GM12878 with *HindIII*, mosquito, and Mediterranean fruit fly used the SMRTbell 3.0 kit. Size-selected libraries were sequenced either on PacBio Sequel II or Revio platforms for 24 hr or 30 hr (Table S1).

### Modifications for cell titration CiFi library preparation

To accommodate samples with limited cell numbers, the standard Hi-C library preparation protocol was proportionally scaled down (Table S6). For inputs of 5 million cells, reagents and volumes were reduced to 50% relative to the original approach for 10 million cells. For inputs between 1 million and 500,000 cells, volumes were reduced to 20%, and for inputs between 250,000 and 62,500 cells, a 10% scale-down was applied. These adjustments enabled efficient library preparation while maintaining protocol integrity.

### Modifications for CiFi library preparation using insect tissue

For a single prepared mosquito *A. coluzzii*, the above-described CiFi protocol was performed with minor modifications in size-selection steps. These included a 2x bead:sample SPRI select purification of following proximity ligation and reverse cross-linking, and 0.45:1 bead:sample ratio of AMPure PB beads prior to SMRTbell library preparation using the SMRTbell 3.0 kit.

To prepare a CiFi library from Mediterranean fruit fly *C. capitata* tissue, the following modifications to the above protocol were made (Table S6). Cross-linking (fixation) was performed using a 2% formaldehyde solution and centrifugations were performed at 1500 × g for 5 minutes. Cross-link reversal was performed using 100 μL 20 mg/mL Proteinase K, 100 μL of 10% SDS, 72 μL of 5M NaCl, and 728 μL NFW and DNA isolated using 1.8x SPRI-select purification. To minimize the presence of small fragments after proximity ligation and cross-link reversal but before gDNA amplification and library preparation, the isolated DNA was size-selected using a 35% dilution of AMPure PB beads at 3.1x, to remove fragments smaller than 5 kbp. After SMRTbell library preparation using the SMRTbell 3.0 kit, the final library was size-selected using the BluePippin to remove fragments smaller than 8 kbp.

### Sequence data processing

The sequence data was processed using the Pore-C workflow^5^ with minor modifications made to account for differences in PacBio versus nanopore reads. Briefly, we performed an *in-silico* digest using the *Pore-c-py* python package to split CiFi concatemer reads at known cut sites of the designated enzyme. The resulting segments were then aligned to a reference genome using minimap2^22^ modified for PacBio reads. A mock “Illumina-like” paired-end BAM was generated from CiFi segments within each HiFi read, representing all possible paired interactions within the HiFi read. A contact matrix was produced using the paired-end BAM file using *pairtools*^23^ and *juicertools*^24^.

### Phased diploid genome assembly of a single insect

To demonstrate the application of the CiFi to genome assembly of samples with limited tissue, a single Mediterranean fruit fly, *Ceratitis capitata*, was used to generate a uniquely barcoded PacBio HiFi low-input SMRTbell WGS library using the SMRTbell 3.0 kit and a uniquely barcoded CiFi library which allows flexibility in sequencing across different SMRT Cells or on one. A single *C. capitata* male was divided laterally where half of the tissue (~10 mg) was prepared into a CiFi library using *HindIII* and the insect CiFi library preparation protocol described above. Its corresponding other half was prepared into a SMRTbell WGS library. These two libraries were sequenced on separate Revio SMRT Cells where the CiFi library was pooled with CiFi libraries prepared for other species (representing about 12.5% of the run) and the WGS library was sequenced in a separate SMRT Cell by itself. A separate single *C. capitata* male was similarly divided where half of the tissue was prepared into a CiFi library using *NlaIII* and its corresponding other half was prepared into a WGS library. These two libraries were sequenced on one Revio SMRT Cell, pooling to approximate 80% of the data coming from the WGS library and 20% from the CiFi library.

The resulting HiFi and CiFi reads were used to create a separate single individual assembly for each CiFi/HiFi combination from each restriction enzyme. A phased assembly of each autosome and an assembly of both sex chromosomes was produced using HiFiASM v.0.24.0-r702 and the `--dual-scaf` option which scaffolds using the graph structure of both phases^13^. Assessment of genome completeness was performed using BUSCO v.5.7.1^16^ to identify the presence of the 3285 genes in the Diptera v10 database (diptera odb10). Raw and adjusted quality values representing base accuracy of the contig assemblies were calculated using YAK^15^. The contig assemblies were scaffolded with the matching individual CiFi reads using the Pore-C bioinformatic pipeline for desegmentation, mapping, and read pairing and YAHS v.1.2a.2 for scaffolding using the resulting read pairs^14^. Contig and scaffold statistics were calculated using the stats.sh function of BBMap^25^. Subsequently, one of the haplotypes of the *HindIII* assembly was scaffolded using a downsampling of the corresponding CiFi reads, from 1,200,000 to 10,000 starting HiFi reads (4,600,000 down to ~40,000 final proximity ligated pairs respectively).

### Statistical analyses

PCR duplication rate was determined using *pbmarkdup*. Read-coverage comparisons were performed using Mosdepth^26^ on the aligned 3C segments using 5 kbp windows with 1 kbp overlap between windows. Data was smoothed using a generalized additive model and the resulting trend lines compared between the standard 3C and CiFi sequence data. Distribution of fragment sizes and number of segments per read for *DpnII* and *HindIII* was quantified using R package *tidyverse*^27^. The visualization of aligned segments of a CiFi read was performed using *SVbyEye*^28^. Published paired-end Illumina reads representing GM12878 *DpnII* Hi-C^10^ were mapped to T2T-CHM13_v2 and processed using *juicertools*^24^. Bedfiles were downloaded from UCSC genome browser representing different repetitive sequences including SDs^29^, interspersed repeats^30^, cenSat^31^, as well as unique sequences. Bedfiles were intersected with aligned CiFi segments using *bedtools*^32^ and percentages of mapped segments were quantified at varying ≥ MAPQ 1, 10, 20, 30, and 60. Chromatin contact matrices were visualized using *Juicebox*^33^ and UCSC genome browser. Chromatin contact counts at 2.5 Mbp and 50 kbp resolutions were extracted from CiFi and Hi-C contact matrices using *juicertools dump* and correlations were calculated using R package *tidyverse*. Topologically-associating domains were called using *TopDom*^34^ and measure of concordance (MoC)^35^ was used to compare between CiFi and Illumina across chromosome 1.

### Notes and considerations

Throughout the protocol, care was taken to prevent DNA shearing by using wide-bore pipette tips and avoiding vortexing. We recommend keeping the samples on ice at all times when not incubating, unless stated otherwise in the protocol. Quality control steps using Qubit assays and Femto Pulse electrophoresis were essential to monitor DNA concentration and fragment size at various stages. Reagent preparation and storage followed manufacturer recommendations to ensure enzyme activity and reaction efficiency. PCR cycle numbers were optimized based on DNA yield to produce sufficient library material for sequencing.

## Supplementary Material

Supplement 1

Supplement 2

## Data Availability

Upon publication, all data will be available through the European Nucleotide Archive and NCBI GenBank through accession PRJEB83708.
