# Supplementary material for: CiFi: Accurate long-read chromatin conformation capture with low-input requirements
Source: bioRxiv. 2025 Feb 5:2025.01.31.635566. Preprint. [Version 1] doi: 10.1101/2025.01.31.635566 (PMC11838532; doi:10.1101/2025.01.31.635566)
Supplement: Supplement 1 [file media-1.pdf]

Supplementary Information

CiFi: Accurate long-read chromatin conformation capture sequencing with low-input requirements

Sean P. McGinty[1*], Gulhan Kaya[1*], Sheina B. Sim[2], Renee L. Corpuz[2], Michael Quail[3], Mara K. N. Lawniczak[3], Scott M. Geib[2], Jonas Korlach[4†], Megan Y. Dennis[1†]

1. Supplementary Figures

Figure S1. Genome-wide sequencing coverage statistics.
Figure S2. Workflow of the CiFi library preparation protocol.
Figure S3. Mapping quality of CiFi and Hi-C reads across the genome.
Figure S4. Comparisons of chromatin contacts for human LCL GM12878 between CiFi and Hi-C.
Figure S5. Correlation of chromatin contacts at 2.5 Mbp resolutions between *DpnII* CiFi and *DpnII* Hi-C.
Figure S6. Topologically-associating domains for GM2878 LCL DpnII CiFi vs HiC across a unique space.
Figure S7. Chromatin contacts for *Anopheles coluzzii* mosquito using *DpnII* CiFi**.**
Figure S8. Scaffolding the Mediterranean fruit fly assembly with CiFi.
Figure S9. Quality check of 3C DNA.
Figure S10. Verification of fragment size during CiFi library preparation.

2. Extended Experimental procedures

I. Detailed CiFi Protocol

Part 1: 3C library preparation
i. Cross-linking and quenching.
ii. Restriction enzyme digestion.
iii. Proximity ligation and reverse cross-linking.
iv. Protein degradation and DNA purification.
Part 2: SMRTbell library preparation from modified ultra-low DNA input
Part 2A: SMRTbell library preparation with Express Template Prep Kit 2.0
i. Removing single-strand overhangs.
ii. Repair DNA damage.
Iii. Repair ends/A-tailing.
iv. Adapter ligation.
v. Purification of SMRTbell library.
vi. Library amplification by modified PCR.
vii. Purification of amplified DNA.
viii. Repair DNA damage.
ix. Repair ends/A-tailing.
x. Adapter ligation.
xi. Purification of SMRTbell library.
xii. BluePippin or diluted AMPure PB bead cleanup, size selection, and sequencing.
Part 2B: SMRTbell library preparation with Prep Kit 3.0
i. Repair and A-tailing of digested 3C DNA.
ii. Ligation of linear amplification adapter and cleanup.
iii. Amplification and cleanup.
iv. Repair and A-tailing of amplified DNA.
v. SMRTbell adapter ligation and cleanup.
vi. Nuclease treatment.
vii. BluePippin or diluted AMPure PB bead cleanup, size selection, and sequencing.

1. Supplementary Figures

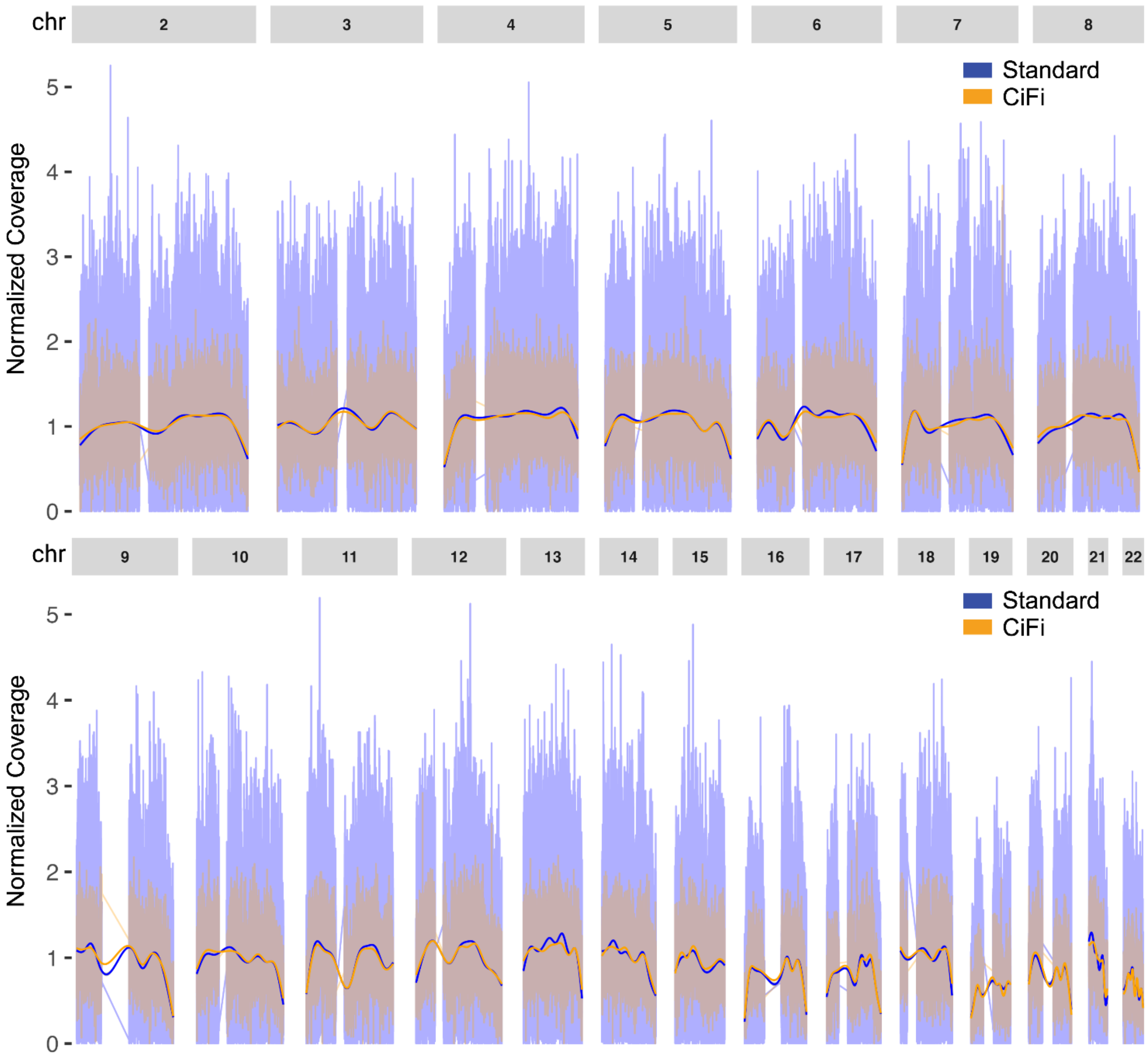

Figure S1. Genome-wide sequencing coverage statistics. Normalized read coverage comparison of Sequel II data for DpnII 3C libraries generated without (Standard) and with the amplification-protocol (CiFi) for GM12878 across human chromosomes 2 through 22

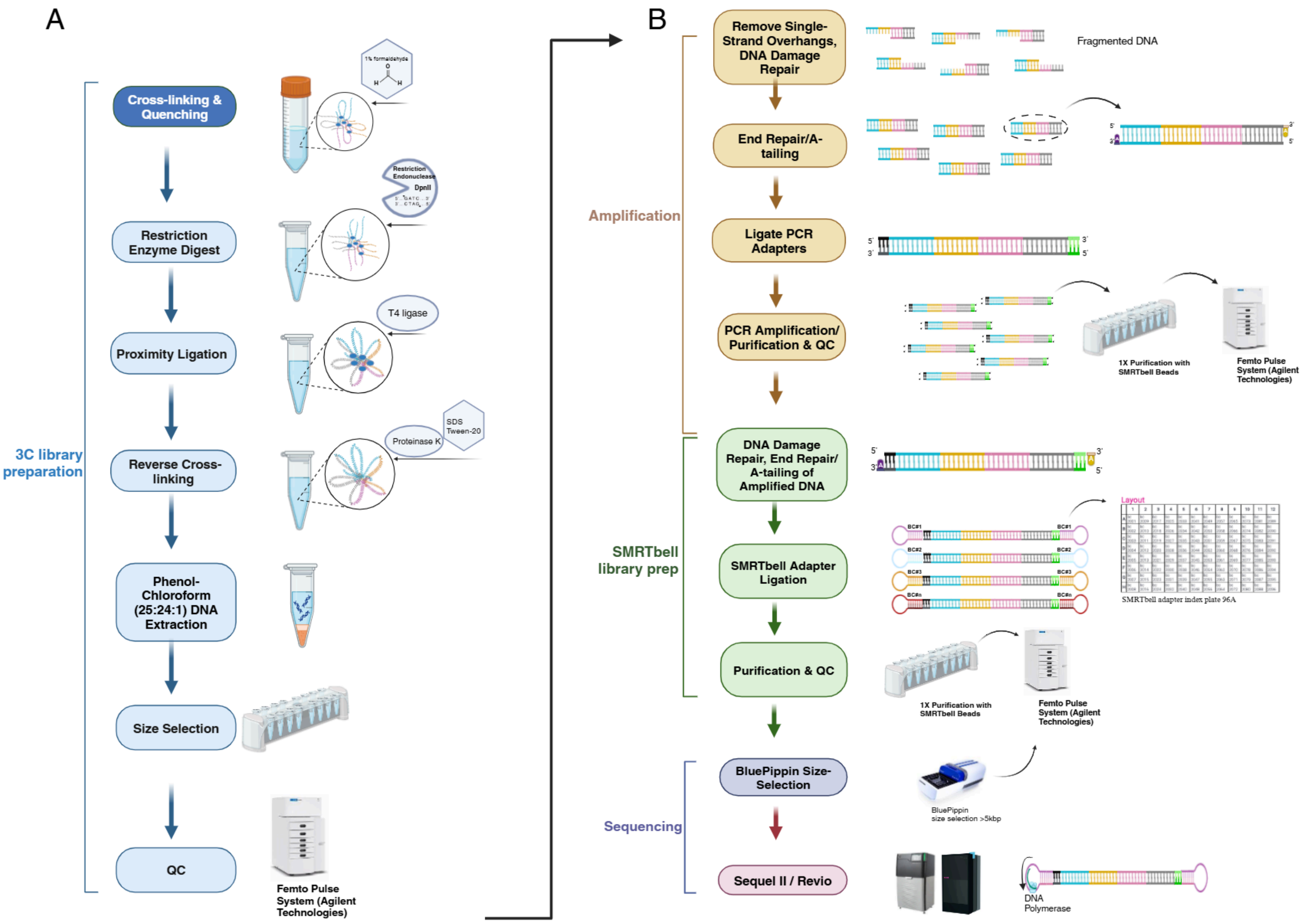

Figure S2. Workflow of the CiFi library preparation protocol. For generating HiFi sequencing libraries from 3C DNA, the workflow is divided into two main parts: **(A)** 3C Library Preparation: Includes cross-linking chromatin, restriction enzyme digestion, proximity ligation, reverse cross-linking, DNA purification, and size selection. **(B)** SMRTbell Library Preparation: Following amplification, DNA damage repair, A-tailing, and adapter ligation are performed. Final libraries undergo size selection using BluePippin and sequencing on the PacBio Sequel II or Revio systems.

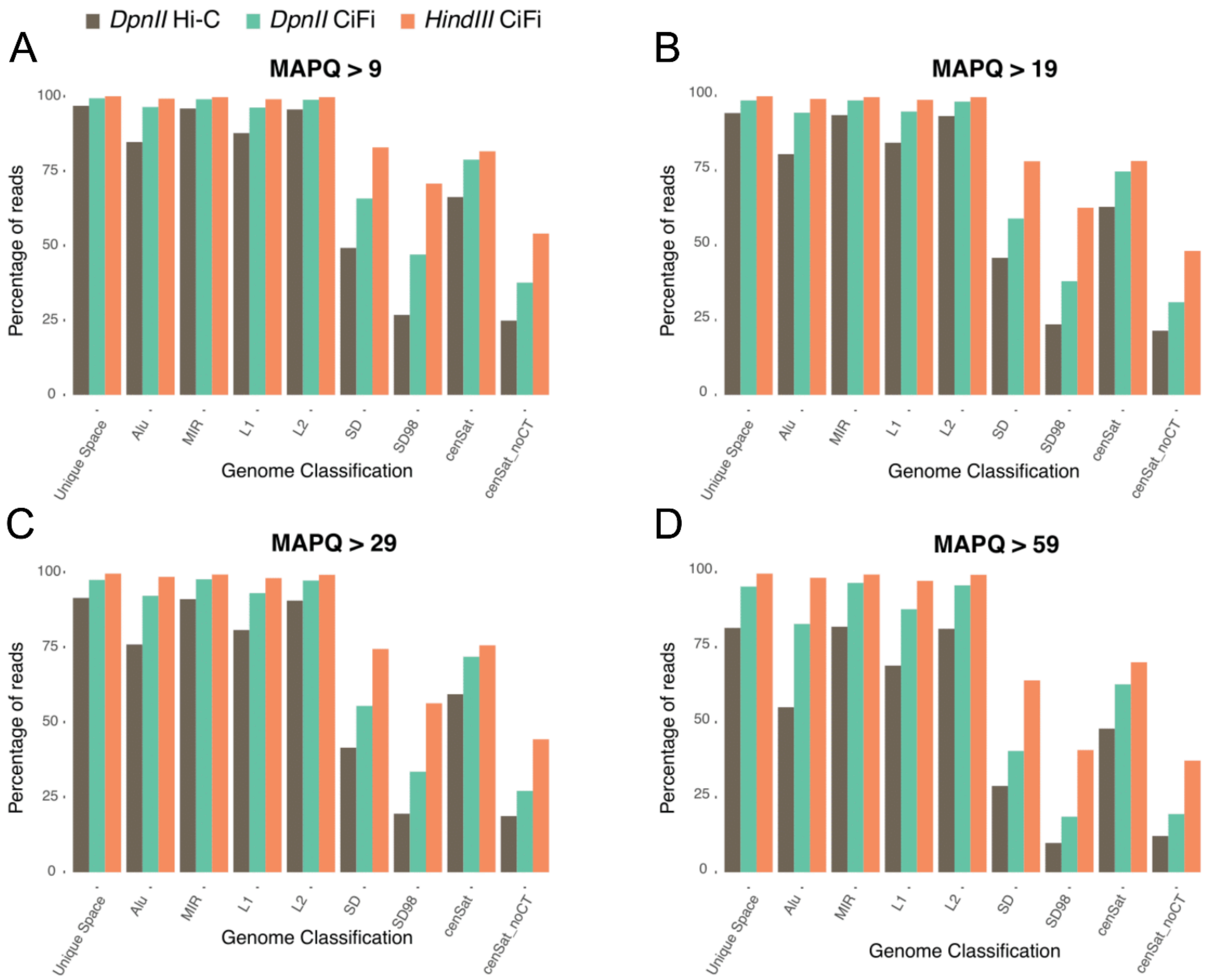

Figure S3. Mapping quality of CiFi and Hi-C reads across the genome. Percentage of reads with varied MAPQ cutoffs (indicated above each plot) for Hi-C with Illumina (Rao et al., 2014) and CiFi across different repetitive genome classifications.

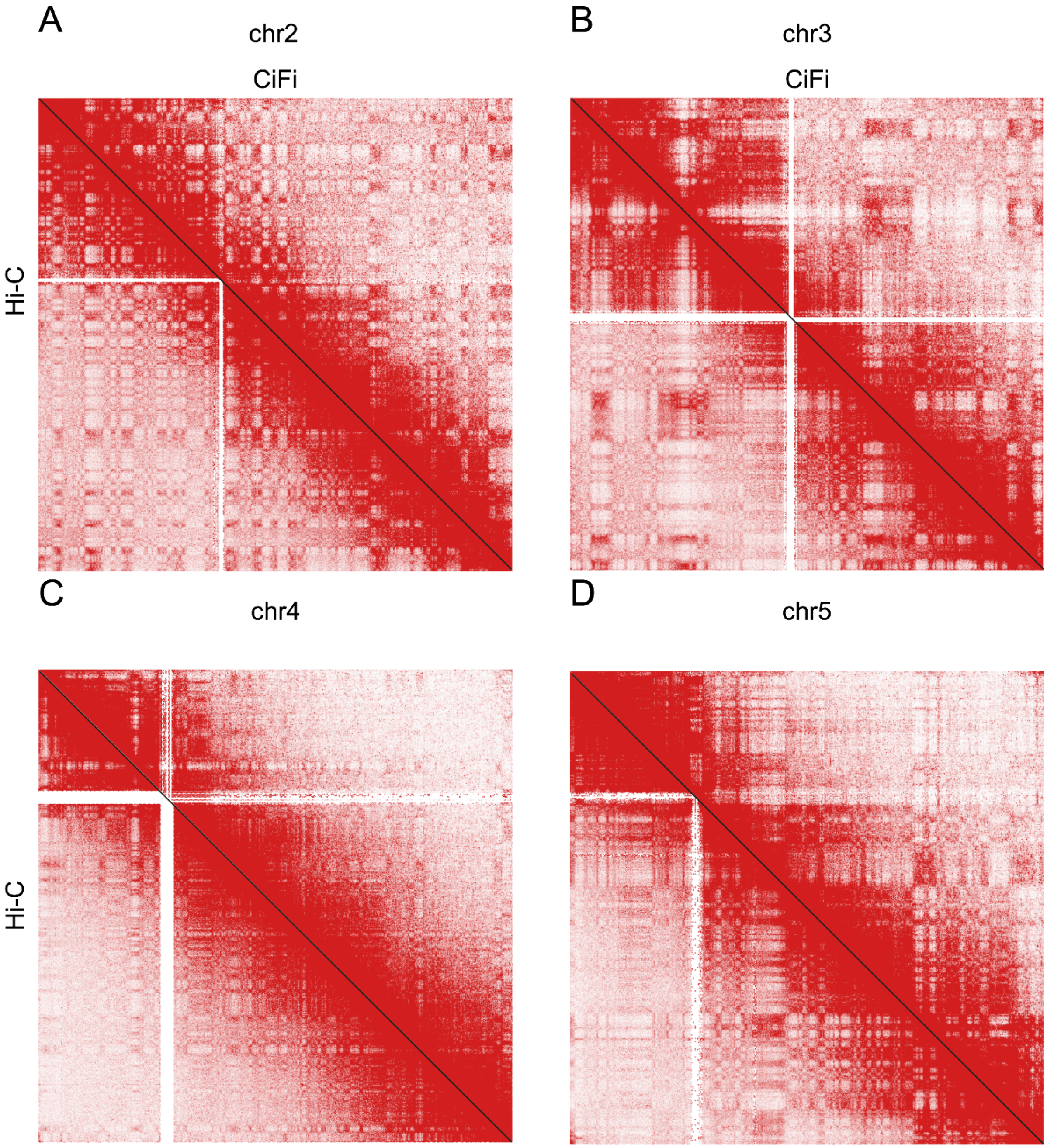

Figure S4. Comparisons of chromatin contacts for human LCL GM12878 between CiFi and Hi-C. Chromosome-scale pairwise interaction maps at 2.5 Mbp resolution for **(A)** chromosome 2, **(B)** chromosome 3, **(C)** chromosome 4, and **(D)** chromosome 5. Red color scales with the number of paired reads per bin. Contact matrices are normalized using Knights-Ruiz algorithm. CiFi is above the diagonal while Hi-C is below.

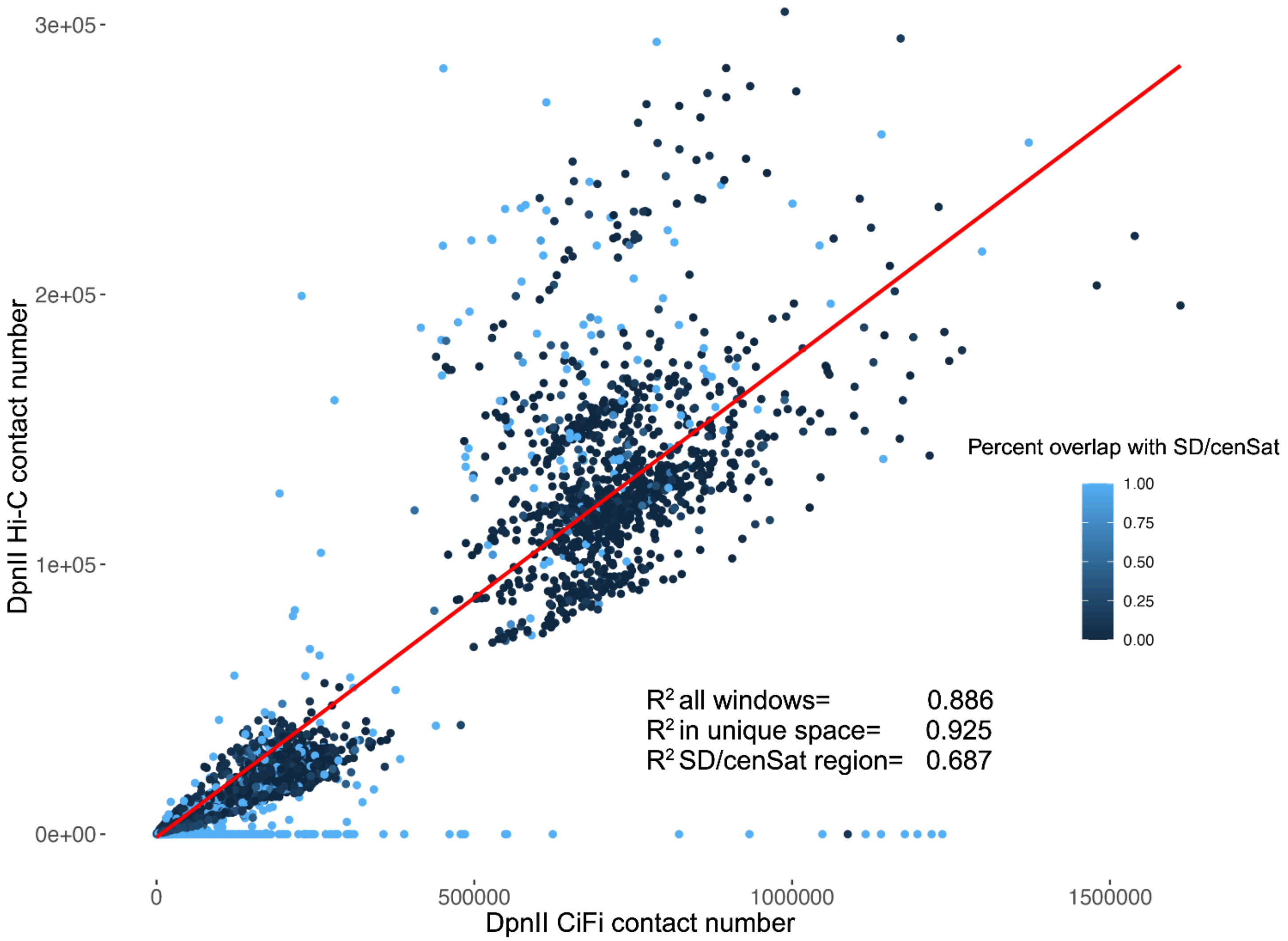

Figure S5. Correlation of chromatin contacts between *DpnII* CiFi and *DpnII* Hi-C. Analyzing the R^2 across all windows at 2.5 Mbp resolution, those in unique space (< 50% overlap with SD/cenSat), or those in SD/cenSat space (>50% overlap), we observe the highest correlation when only accounting for windows in unique space and the lowest when looking at windows that overlap SD/cenSat. This discordance shows that CiFi leads to a gain of contacts across repetitive regions.

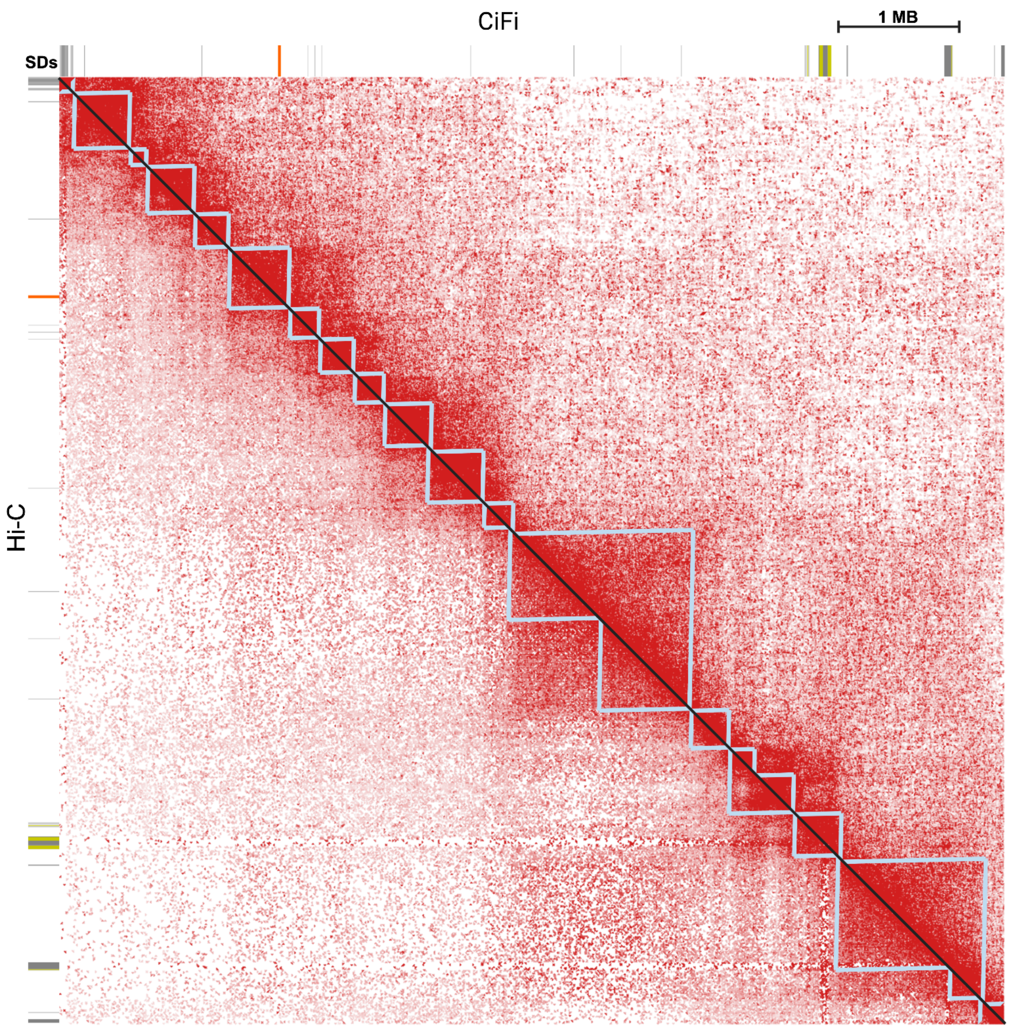

Figure S6. Topologically-associating domains for GM2878 LCL *DpnII* CiFi vs HiC across a unique space. Human chromosome 2 (chr2:98,000,000-109,000,000, T2T-CHM1_v2; 50 kbp resolution), contacts are normalized using Knights-Ruiz algorithm. Topologically-associating domains are represented as yellow triangles. CiFi is above the diagonal while Hi-C is below.

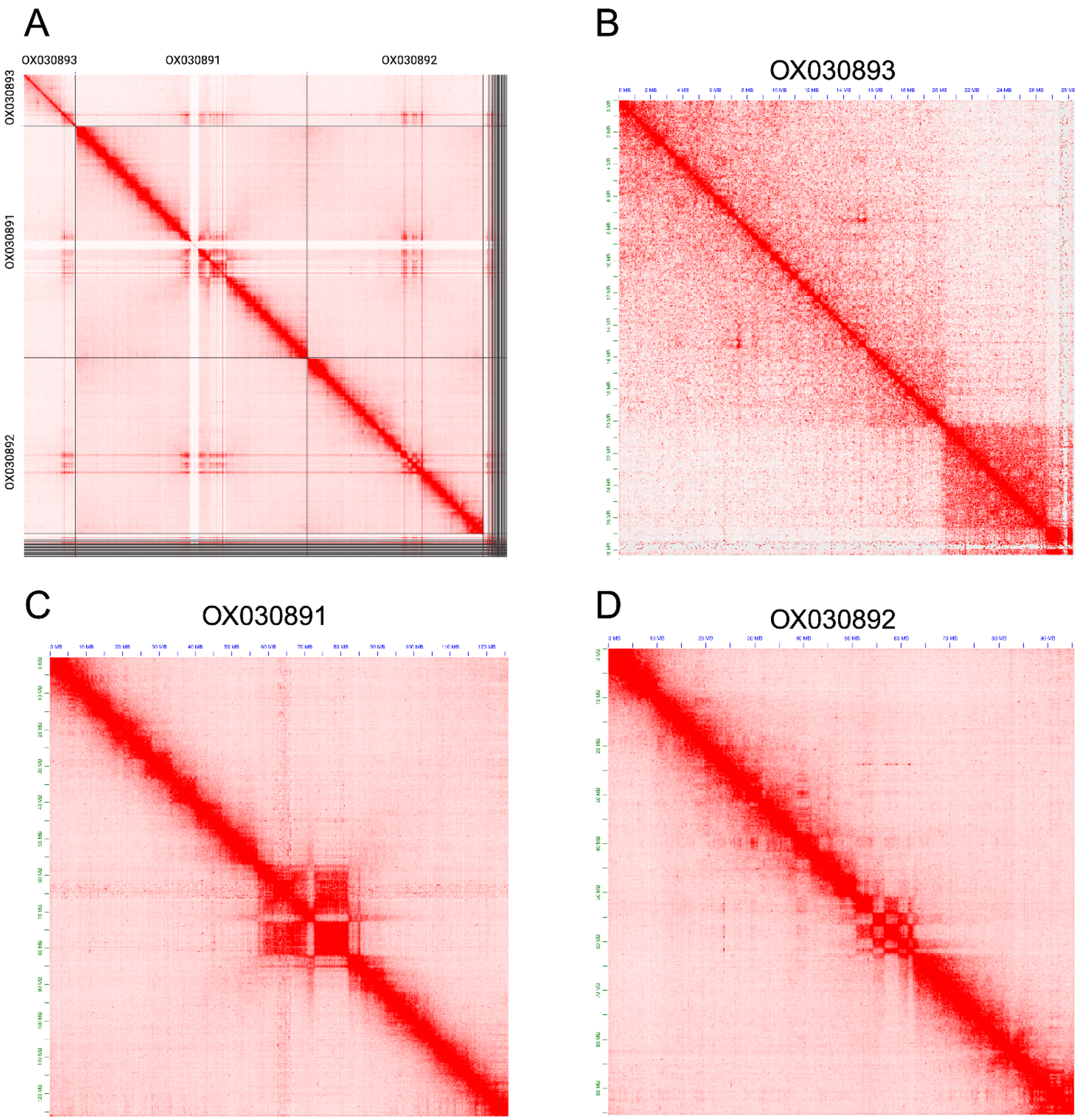

Figure S7. Chromatin contacts for *Anopheles coluzzii* mosquito using *DpnII* CiFi. Pairwise interactions maps of data from an Ngousso colony individual mapped to the 263 Mbp reference genome AcolN3 (created from the same colony but some years before) quantified **(A)** genome wide and across **(B)** chromosome OX030893 at 50 kbp resolution, **(C)** chromosome OX030891 at 250 Kbp resolution, and **(D)** chromosome OX030892 at 250 kbp resolution. Chromosome scale contact matrices were normalized using the Vanilla-Coverage (VC) algorithm.

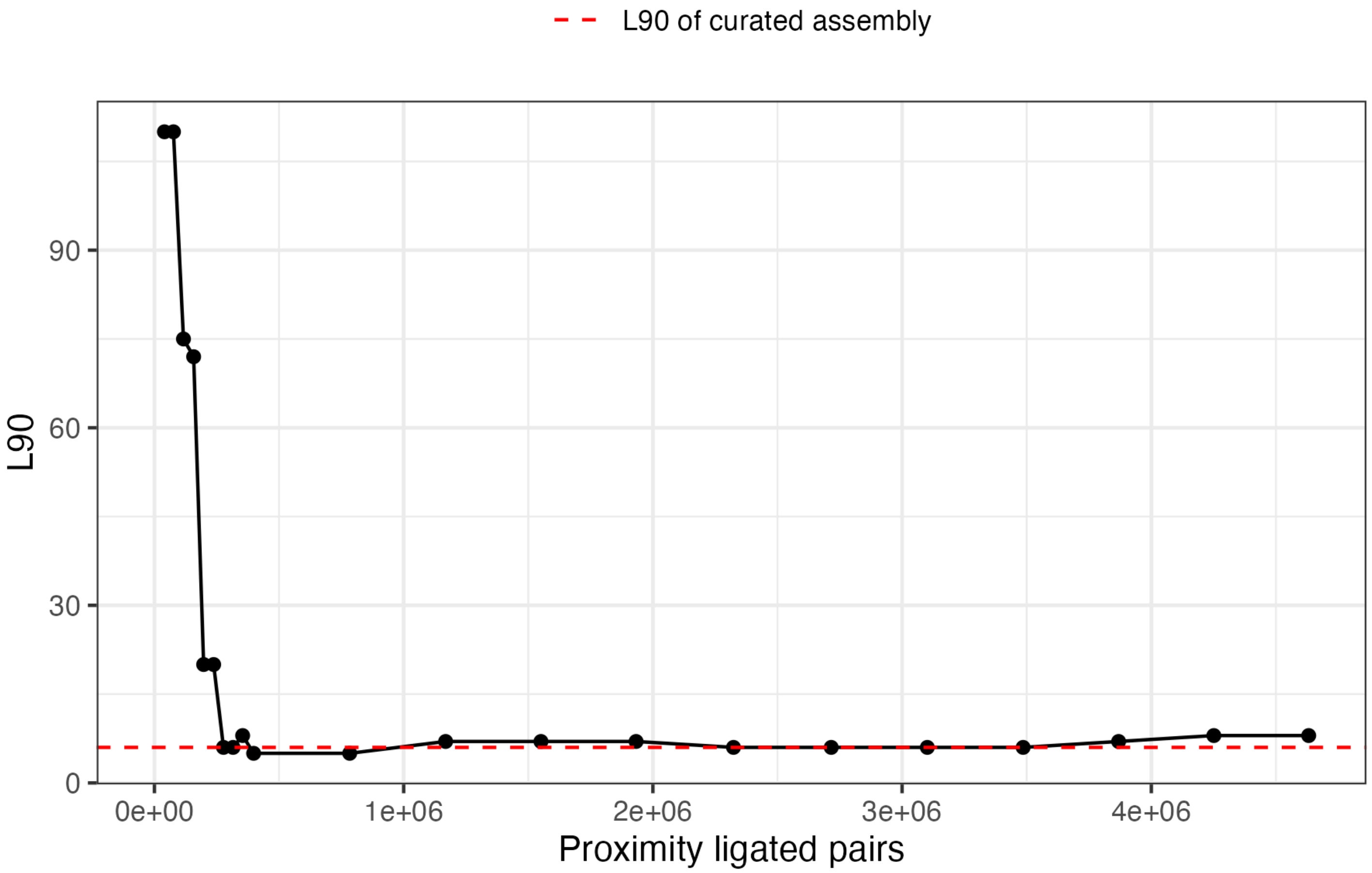

Figure S8. Scaffolding the Mediterranean fruit fly assembly with CiFi. The number of proximity ligated pairs from the *HindIII* CiFi library necessary to scaffold a *C. capitata* genome. For this 600 Mbp phased genome, ~300,000 proximity ligated pairs from 70,000 CiFi reads (~1.5x coverage of the genome) was necessary for scaffolding the phased assembly. Dashed red line denoting the L90 of haplotype one of the curated reference (Papanicolaou et al. 2016) is shown for reference.

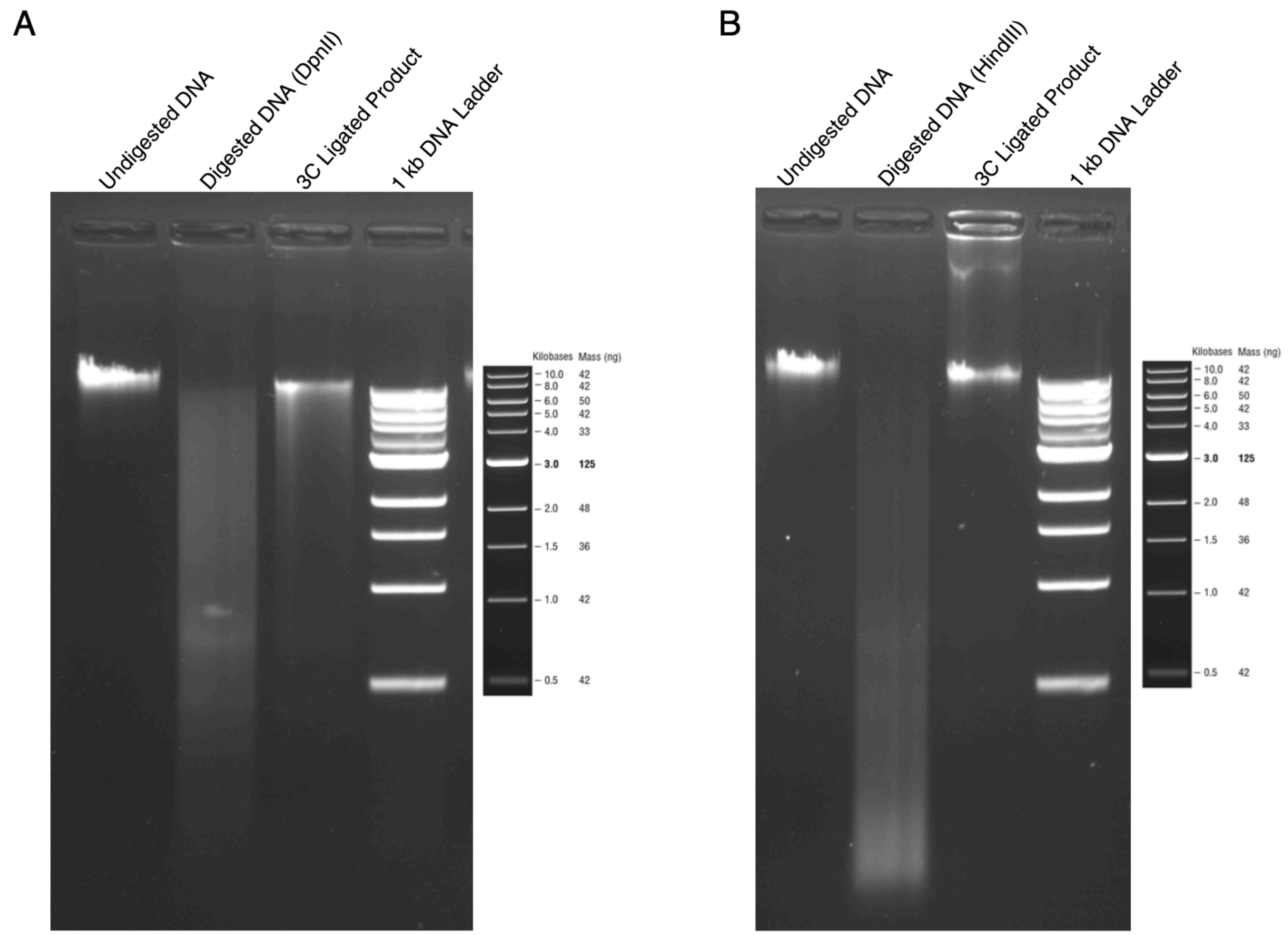

Figure S9. Quality check of 3C DNA. 3C DNA obtained from GM12878 cells was analyzed on a 1% agarose gel alongside undigested and digested DNA samples for **(A)** *DpnII* and **(B)** *HindIII* accompanied with a 1 kbp DNA ladder as a molecular weight marker.

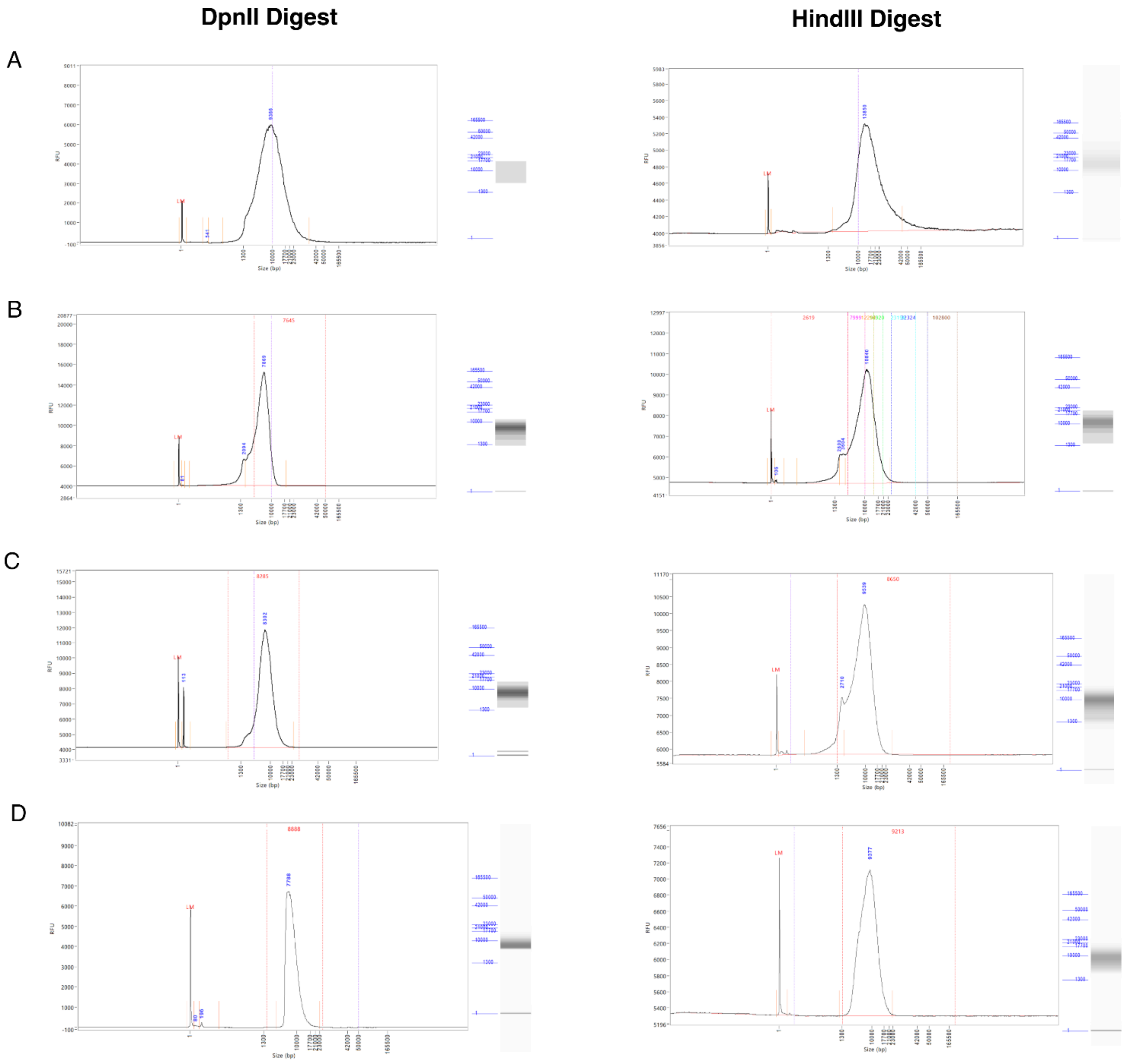

Figure S10. Verification of fragment size during CiFi library preparation. Fragment size distributions were analyzed at key stages of the CiFi library preparation protocol using the Femto Pulse system (Agilent Technologies) for both *DpnII* (left column) and *HindIII* (right column) digests. The rows correspond to the following steps in the protocol: **(A)** 3C DNA QC, (**B)** After amplification, **(C)** After SMRTbell library preparation, and (**D)** After BluePippin size selection.

2. Extended Experimental Procedures

I. Detailed CiFi Protocol

In this study, we report the development and results of the CiFi workflow, designed to capture high-resolution chromatin interactions using HiFi SMRTbell libraries prepared from ultra-low DNA input. The optimized protocol integrates chromatin conformation capture (Hi-C) with PacBio's SMRTbell sequencing, enabling efficient library preparation for long-read sequencing from limited DNA quantities. A detailed step-by-step protocol is provided below.

Part 1: 3C library preparation

Note: Please refer to Table S6 for details on lower cell inputs.

We recommend keeping the samples on ice at all times when not incubating, unless stated otherwise in the protocol, and using wide-bore tips throughout the procedure.

i. Cross-linking and quenching

1. **Preparation**:
   - Wash 5-10 million cells three times in chilled 1X phosphate buffered saline (PBS) in a 50 mL centrifuge tube.
   - Centrifuge at 500xg for 5 minutes at 4°C between each wash.
   - Resuspend cells in 10 mL room temperature 1X PBS with 1% formaldehyde [EMD Millipore cat no. 818708] by gently pipetting **with a wide bore tip.**
   - Incubate at room temperature for 10 minutes.
2. **Quenching**:
   - Add 527 µL of 2.5 M glycine to achieve a final concentration of 1% (125 mM) in 10.5 mL.
   - Incubate for 5 minutes at room temperature followed by 10 minutes on ice.
   - Pellet the cross-linked cells by centrifugation at 500xg for 5 minutes at 4°C.
   - Wash the cross-linked cells with 1x PBS, remove the supernatant, and snap-freeze the pellet in liquid nitrogen. Store at -80°C until you start the protocol.

ii. Restriction enzyme digestion

1. **Cell lysis**:
   - Resuspend the cell pellet in a mixture of 50 µL of protease inhibitor cocktail [Sigma Aldrich cat no. P8340] in 500 µL of cold permeabilization buffer (10 mM Tris-HCl pH 8.0, 10 mM NaCl, 0.2% IGEPAL CA-630).
   - Place on ice for 15 minutes.
   - Centrifuge at 500xg for 10 minutes at 4°C.
   - Aspirate the supernatant and replace with 200 µL of chilled 1.5X digestion reaction buffer [NEB] compatible with the restriction enzyme used.
   - Centrifuge again at 500xg for 10 minutes at 4°C, then aspirate and resuspend in 300 µL of chilled 1.5X digestion reaction buffer.

2. **Chromatin denaturation**:
   - Add 33.5 µL of 1% w/v SDS [Thermo Fisher Scientific cat no. 15553027] to each cell suspension.
   - Incubate for exactly 10 minutes at 65°C with gentle agitation.
   - Place on ice immediately afterwards.

- Quench the SDS by adding 37.5 µL of 10% v/v Triton X-100 [Sigma Aldrich cat no. 93443] for a final concentration of 1%.
- Incubate for 10 minutes on ice.

3. **Digestion**:
   - Permeabilized cells are then digested with a final concentration of 1 U/µL of *DpnII* [NEB], brought to volume with nuclease-free water to achieve a final 1X digestion reaction buffer in 450 µL.
   - Mix by gentle inversion and incubate in a thermomixer at 37°C for 18 hours with periodic < 1000 rpm rotation (< 30 seconds every 15 minutes) to prevent condensation inside the lid.

iii. Proximity ligation and reverse cross-linking

1. **Inactivation**:
   - *DpnII* restriction digests are heat-inactivated at 65°C for 20 minutes with 300 rpm rotation. Place on ice immediately afterwards.
2. **Ligation**:
   - Set up proximity ligation at room temperature with the following reagents:
     - 100 µL of 10X T4 DNA ligase buffer [NEB]
     - 10 µL of 10 mg/mL BSA
     - 50 µL of T4 DNA ligase [NEB M0202L]
     - **Total volume of 1000 µL** with nuclease-free water.
   - Cool to 16°C and incubate for 6 hours with gentle rotation.

iv. Protein degradation and DNA purification

1. **Reverse cross-linking**:
   - Treat samples with:
     - 100 µL 20 mg/mL Proteinase K [Thermo Fisher Scientific cat no. 25530049]
     - 100 µL 10% SDS [Thermo Fisher Scientific cat no. 15553027]
     - 500 µL 20% v/v Tween-20 [Sigma Aldrich cat no. P9416]
     - **Total volume of 2000 µL** with nuclease-free water.
   - Incubate in a thermomixer at 56°C for 18 hours with < 1000 rpm rotation (< 30 seconds every 15 minutes) to prevent condensation inside the lid.

2. **Purification**:
   - Transfer the sample to a 15 mL centrifuge tube, rinsing the original tube with a further 200 µL of nuclease-free H2O to collect any residual sample, bringing the total sample volume to 2.2 mL.
   - Purify DNA using standard phenol-chloroform extraction and ethanol precipitation method.
   - Before proceeding, check undigested, digested, and ligated DNA products on an agarose gel to ensure the experiment was successful (Figure S9).
   - Store the purified libraries at 4°C for short-term storage, and at -20°C for long-term storage until the next step.

Size selection (AMPure PB bead, 0.45x)

Follow the PacBio protocol for size selection using PacBio AMPure PB beads (PacBio, 100-265-900) at a 0.45× ratio. Add 50 µL of TE to resuspend the beads, then proceed to the next step.

- For libraries prepared using ***DpnII***, proceed directly to size selection.
- For libraries prepared using ***HindIII***, the longer DNA fragments require an additional shearing step before size selection. Shear the 3C DNA to approximately 10 kb using a g-TUBE (Covaris, 520104), following the manufacturer's protocol.
- After size selection, use 0.5 ng/µL of DNA for quality control (QC) with Femto Pulse automated pulsed-field capillary electrophoresis to confirm that the DNA matches the expected size distribution (Figure S10A)

Part 2: SMRTbell library preparation from modified ultra-low DNA input

Required PacBio kits:
SMRTbell gDNA amplification kit, cat# 101-980-000
SMRTbell adapter index plate 96A, cat# 102-009-200
SMRTbell cleanup beads, cat# 102-158-300
SMRTbell express template prep kit 2.0, cat# 100-938-900*
Alternative Kit: SMRTbell prep kit 3.0, cat# 102-182-700*

*Note: The initial optimization of the CiFi protocol for GM12878 with *DpnII* used the SMRTbell Express Template Prep Kit 2.0, with the detailed protocol described below in Part 2A. CiFi libraries for GM12878 with *HindIII*, mosquito, and Mediterranean fruit fly used the SMRTbell Prep Kit 3.0, with the detailed protocol provided in Part 2B.

PCR amplification:
KOD Xtreme hot-start polymerase from Sigma, cat# 71975-M

PacBio library construction requirements for ultra-low DNA input samples:

Required gDNA Input Amount	Required Quality of Input gDNA	gDNA Shearing Method	Target Sheared Fragment Size Distribution Mode	Amplification Target Size Distribution Mode	Total Mass of Pooled PCR Product Required for Library Construction	Required SMRTbell Library Input for BluePippin Size-Selection
5-20 ng	Majority of gDNA >20 kb	Megaruptor or g-TUBE	10 kb sheared DNA is optimal	8-10 kb	≥500 ng	≥400ng

Note: Skip the gDNA shearing step for *DpnII* since we already digested it with the restriction enzyme, perform only for *HindIII* libraries (as described above).

Part 2A: SMRTbell library preparation with Express Template Prep Kit 2.0

i. Removing single-strand overhangs

1. **Preparation of DNA prep additive:**
    - Dilute the DNA prep additive with enzyme dilution buffer. Mix well and quick spin.
        - 4.0 µL Enzyme dilution buffer
        - 1.0 µL DNA prep additive (stock)
        - **Total volume: 5 µL**
2. **Reaction mix preparation:** For each sample, prepare the following reaction mix in a PCR tube:
    - 7.0 µL DNA prep buffer
    - <45 µL 3C DNA
    - 0.6 µL NAD
    - 1.0 µL Diluted DNA prep additive (prepared above)
    - 1.0 µL DNA prep enzyme
    - H_2O up to a **total volume of 55.0 µL**

3. Mix thoroughly using wide-bore pipette tips (pipette mix 10 times).
4. Quick spin to collect contents in a microfuge.
5. Place in a thermocycler and run the following program:
    - 15 minutes at 37°C
    - Hold at 4°C
6. Proceed to the next step.

ii. Repair DNA damage

1. **Reaction setup:** Add 2 µL DNA damage repair mix v2 directly to the reaction:
    - 55.0 µL Reaction mix
    - 2.0 µL DNA damage repair mix v2
    - **Total volume: 57.0 µL**
2. Mix thoroughly using wide-bore pipette tips (pipette mix 10 times).
3. Quick spin to collect contents in a microfuge.
4. Place in a thermocycler and run the following program:
    - 30 minutes at 37°C
    - Hold at 4°C
5. Proceed to the next step.

iii. Repair ends/A-tailing

1. **Reaction setup:** Add 3 µL end prep mix directly to the reaction:
    - 57.0 µL Reaction mix (damage-repaired sample)
    - 3.0 µL End prep mix
    - **Total volume: 60.0 µL**
2. Mix thoroughly using wide-bore pipette tips (pipette mix 10 times).
3. Quick spin to collect contents in a microfuge.
4. Place in a thermocycler and run the following program:
    - 30 minutes at 20°C
    - 30 minutes at 65°C
    - Hold at 4°C
5. Proceed to the next step.

iv. Adapter ligation

1. **Preparation of amplification adapters:** Dilute the Amplification Adapters with Duplex Buffer:
    - 9.0 µL Duplex Buffer
    - 1.0 µL Amplification Adapters
    - **Total volume: 10.0 µL** Use immediately.
2. **Reaction setup:** Add the following components to the reaction:
    - 60.0 µL Reaction mix (A-tail sample)
    - 2.5 µL Diluted amplification adapters
    - 30.0 µL Ligation mix
    - 1.0 µL Ligation additive
    - 1.0 µL Ligation enhancer
    - **Total volume: 94.5 µL**
3. Mix thoroughly using wide-bore pipette tips (pipette mix 10 times).
4. Quick spin to collect contents in a microfuge.

5. Place in a thermocycler and run the following program:
    - 60 minutes at 20°C
    - Hold at 4°C
6. Proceed to the next step.

v. Purification of SMRTbell library

1. Bring SMRTbell beads (PacBio) to room temperature for 30 to 60 minutes before use.
2. Add 77.5 µL SMRTbell beads to 94.5 µL Reaction Mix 4. Mix gently (pipette mix 10 times).
3. Incubate on the bench for 5 minutes at room temperature.
4. Place the tube on a magnetic stand and wait until the supernatant is clear. Remove the supernatant.
5. Wash beads twice with 200 µL freshly prepared 80% ethanol.
6. After the second ethanol wash, spin briefly, return to the magnetic stand, and remove residual ethanol. Do not let the beads dry out.
7. Resuspend beads in 97 µL of EB, pipette mix 10 times, and incubate at 37°C for 10 minutes to elute DNA.
8. Place on a magnetic stand to separate beads. Transfer 97 µL of purified sample to a new tube and set aside on ice.

IMPORTANT: For the Library Amplification by PCR, the 97 µL of the purified eluted sample will be divided and used for three reactions to achieve enough output product for the next step. Each reaction requires a volume of 32 µL of the purified eluted sample. Sometimes, we need to repeat the PCR reaction 2x or 3x to achieve 1 µg of output.

vi. Library amplification by modified PCR

1. **Reaction setup:** For each reaction:
    - 60 µL 2X Xtreme buffer
    - 24 µL 2 mM dNTPs
    - 2.4 µL Sample amplification PCR primer
    - 32 µL DNA
    - 2.4 µL KOD Xtreme Hot Start DNA polymerase

    Total: 120.8 µL
2. **Thermal cycler settings:**
    - Initial Denaturation: 94°C for 2 minutes (1 cycle)
    - Denaturation: 98°C for 10 seconds (13 cycles)
    - Annealing: 60°C for 30 seconds (13 cycles)
    - Extension: 68°C for 10 minutes (13 cycles)
    - Final Extension: 68°C for 5 minutes (1 cycle)
    - Hold: 4°C indefinitely

vii. Purification of amplified DNA

1. Bring SMRTbell beads to room temperature for 30 to 60 minutes before use.
2. Add 99 µL SMRTbell beads to the 120.8 µL reaction mix. Mix gently (pipette mix 10 times).
3. Incubate on the bench for 5 minutes at room temperature.
4. Place the tube on a magnetic stand and wait until the supernatant is clear. Remove the supernatant.
5. Wash beads twice with 200 µL freshly prepared 80% ethanol.
6. After the second ethanol wash, spin briefly, return to the magnetic stand, and remove residual ethanol. Do not let the beads dry out.

7. Resuspend beads in 26 µL of EB, pipette mix 10 times, and incubate at room temperature for 5 minutes to elute DNA.
8. Place on a magnetic stand to separate beads. Transfer 26 µL of eluted amplified DNA to a new tube and set aside on ice.
9. Use 1 µL of the sample to quantify with Qubit dsDNA HS kit.
10. Use 1 µL of amplified DNA for DNA sizing QC by Femto Pulse automated pulsed-field capillary electrophoresis (200-500 pg of sample) (Figure S10B).
11. Store amplified DNA at 4°C or -20°C for future use.

Total DNA requirement:

- Ensure a pooled DNA mass ≥500 ng in 47.4 µL (recommended: 1 µg).

viii. Repair DNA damage (Post-amplification)

1. **Reaction Setup:** Add the following components to a single PCR tube:
    - 7.0 µL DNA Prep Buffer
    - ≤47.4 µL Pooled Amplified DNA
    - 0.6 µL NAD
    - 2.0 µL DNA Damage Repair Mix v2
    - H_2O up to a **total volume of 57.0 µL**
2. Mix thoroughly using wide-bore pipette tips (pipette mix 10 times).
3. Quick spin to collect contents in a microfuge.
4. Place in a thermocycler and run the following program:
    - 30 minutes at 37°C
    - Hold at 4°C
5. Proceed to the next step.

ix. Repair ends/A-tailing

1. **Reaction setup:** Add 3 µL End Prep Mix directly to the reaction:
    - 57.0 µL Reaction mix (damage-repaired sample)
    - 3.0 µL End prep mix
    - **Total volume: 60.0 µL**
2. Mix thoroughly using wide-bore pipette tips (pipette mix 10 times).
3. Quick spin to collect contents in a microfuge.
4. Place in a thermocycler and run the following program:
    - 30 minutes at 20°C
    - 30 minutes at 65°C
    - Hold at 4°C
5. Proceed to the next step.

x. Adapter ligation

1. **Reaction setup:** Add the following components to Reaction Mix 7 (A-tail sample):
    - 60.0 µL Reaction mix
    - 5.0 µL Overhang adapter v3 (or SMRTbell adapter index plate for barcoding)
    - 30.0 µL Ligation mix
    - 1.0 µL Ligation additive

- 1.0 µL Ligation enhancer
    - **Total volume: 97 µL**
2. Mix thoroughly using wide-bore pipette tips (pipette mix 10 times).
3. Quick spin to collect contents in a microfuge.
4. Place in a thermocycler and run the following program:
    - 60 minutes at 20°C
    - Hold at 4°C
5. Proceed to the next step: "Purification of SMRTbell Library."

xi. Purification of SMRTbell library

1. Add 97 µL of room-temperature resuspended SMRTbell beads to 97 µL reaction mix. Pipette mix 10 times.
2. Perform a quick spin to collect liquid.
3. Incubate on the bench for 5 minutes at room temperature.
4. Place on a magnetic stand to separate beads from supernatant. Remove supernatant using a P200 pipettor.
5. Wash beads twice with 200 µL freshly prepared 80% ethanol. After the second wash, briefly spin and return to the magnet. Remove residual ethanol using a P20 pipette.
6. Resuspend beads in 32 µL EB, mix thoroughly, and incubate at room temperature for 5 minutes to elute DNA.
7. Place on the magnetic stand to separate beads. Transfer eluted DNA to a new tube.
8. Use 1 µL of SMRTbell library to quantify with Qubit dsDNA HS kit.
9. Use 1 µL for DNA sizing QC by Femto Pulse (200-500 pg sample) (Figure S10C).
10. Proceed with "BluePippin Size-Selection of SMRTbell Library" or store at 4°C or -20°C.

xii. BluePippin or diluted AMPure PB bead cleanup, size selection, and sequencing

For size selection of SMRTbell libraries, you can use either BluePippin or diluted AMPure PB beads based on the desired fragment size and available resources. Both methods enrich DNA fragments within specific size ranges and are compatible with sequencing on the Revio or Sequel II platforms.

- BluePippin size selection:
Perform >5 kb size selection using BluePippin according to the manufacturer's protocol.

- Alternatively, diluted AMPure PB bead cleanup and size selection:
Perform ~3 kb size selection using diluted AMPure PB beads to enrich the sample for DNA fragments of the desired size.

Instructions for diluted AMPure PB bead preparation and use:

- **Prepare 35% AMPure PB beads:**
  Mix 1.75 mL of resuspended AMPure PB beads with 3.25 mL of elution buffer (35% v/v).
  Store the diluted beads at 4°C for up to 30 days.
- **Size selection procedure:**
    1. Add 3.1X volume of diluted beads to the sample.
    2. Mix thoroughly and briefly spin down.
    3. Incubate at room temperature for 10 minutes to bind DNA.
    4. Place on a magnetic rack and remove the supernatant.
    5. Wash beads twice with 200 µL of 80% ethanol.
    6. Air-dry the beads and resuspend in 50 µL of elution buffer.

7. Incubate for 5 minutes and use the magnetic rack to separate the beads.
8. Transfer the supernatant containing the size-selected DNA to a new tube for sequencing or storage.

After size selection, use 1 µL of the sample for library sizing QC by Femto Pulse (200-500 pg sample) (Figure S10D)

Part 2B: SMRTbell library preparation with Prep Kit 3.0

i. Repair and A-tailing of digested 3C DNA

1. **Reaction setup: Add the following components to a PCR tube:**
   - 4 µL Repair buffer
   - 1 µL End repair mix
   - 0.5 µL DNA repair mix
   - 24.5 µL Digested 3C DNA (5-20 ng)
   - **Total volume: 30 µL**
2. Quick spin to collect contents in the tube.
3. Place in a thermocycler and run the following program:
   - 30 minutes at 37°C
   - 5 minutes at 65°C
   - Hold at 4°C
4. Proceed to the next step.

ii. Ligation of linear amplification adapter and cleanup

1. Reaction setup: Add the following components to a PCR tube:
   - 2 µL Amplification adapter
   - 10 µL Ligation mix
   - 0.5 µL Ligation enhancer
   - 30 µL End-prepared DNA
   - **Total volume: 42.5 µL**
2. Quick spin to collect contents in the tube.
3. Place in a thermocycler and run the following program:
   - 30 minutes at 20°C
   - Hold at 4°C
4. Perform cleanup using 1X SMRTbell cleanup beads as described in Part 2A. Add 24 µL of elution buffer to resuspend the beads, then proceed to the next step.

iii. Amplification and cleanup

1. Reaction setup: Add the following components to a PCR tube:
   - 50 µL 2X Xtreme buffer
   - 20 µL 2 mM dNTPs
   - 4 µL Sample amplification PCR primer
   - 2 µL KOD Xtreme Hot Start DNA polymerase
   - 24 µL Adapter-ligated sample
   - **Total volume: 100 µL**
2. Quick spin to collect contents in the tube.
3. Place in a thermocycler and run the following program:
   - Initial Denaturation: 94°C for 2 minutes (1 cycle)

    - Denaturation: 98°C for 10 seconds (10–12 cycles)
    - Annealing and Extension: 68°C for 10 minutes (10–12 cycles)
    - Final Extension: 68°C for 7 minutes (1 cycle)
    - Hold: 4°C indefinitely

DNA input and PCR cycles:

- 5 ng: 12 cycles
- 10 ng: 11 cycles
- 20 ng: 10 cycles

4. Perform cleanup using 1X SMRTbell cleanup beads as described in Part 2A. Add 50 µL of elution buffer to resuspend the beads. Quantify the DNA using the Qubit dsDNA HS Kit and check quality control (QC) using a Femto Pulse (Figure S10B). Proceed to the next step.

Total DNA requirement:

- ≥500 ng of amplified DNA for Revio sequencing.
- ≥250 ng of amplified DNA for Sequel II/IIe sequencing

iv. Repair and A-tailing of amplified DNA

1. Reaction setup: Add the following components to a PCR tube:
    - 8 µL Repair buffer
    - 2 µL End repair mix
    - 1 µL DNA repair mix
    - 49 µL Amplified DNA
    - **Total volume: 60 µL**
2. Quick spin to collect contents in the tube.
3. Place in a thermocycler and run the following program:
    - 30 minutes at 37°C
    - 5 minutes at 65°C
    - Hold at 4°C

v. SMRTbell adapter ligation and cleanup

1. Reaction setup: Add the following components to a PCR tube:
    - 4 µL SMRTbell adapter (or SMRTbell adapter index plate for barcoding)
    - 15 µL Ligation mix
    - 1 µL Ligation enhancer
    - 60 µL Repaired DNA
    - **Total volume: 80 µL**
2. Quick spin to collect contents in the tube.
3. Place in a thermocycler and run the following program:
    - 30 minutes at 20°C
    - Hold at 4°C
4. Perform cleanup using 1X SMRTbell cleanup beads as described in Part 2A. Add 40 µL of elution buffer to resuspend the beads, then proceed to the next step.

vi. Nuclease treatment

1. Reaction Setup: Add the following components to a PCR tube:
    - 5 µL Nuclease buffer
    - 5 µL Nuclease mix
    - 40 µL Ligated DNA
    - **Total volume: 50 µL**
2. Place in a thermocycler and run the following program:
    - 15 minutes at 37°C
    - Hold at 4°C
3. Perform cleanup using 1X SMRTbell cleanup beads as described in Part 2A.

vii. BluePippin or diluted AMPure PB bead cleanup, size selection, and sequencing

As described in the previous section, Part 2A-xii, the procedure was followed to complete this step.
